# Relocation to Activity-Based Workplaces (ABW)—Importance of the Implementation Process

**DOI:** 10.3390/ijerph182111456

**Published:** 2021-10-30

**Authors:** Eva L. Bergsten, Katarina Wijk, David M. Hallman

**Affiliations:** 1 Faculty of Health and Occupational Studies, Department of Occupational Health Sciences and Psychology, University of Gavle, 801 76 Gävle, Sweden; katarina.wijk@regiongavleborg.se (K.W.); david.hallman@hig.se (D.M.H.); 2 Centre for Research and Development, Region Gavleborg/Uppsala University, 801 87 Gävle, Sweden; 3 Department of Public Health and Caring Sciences, Uppsala University, 751 23 Uppsala, Sweden

**Keywords:** activity-based flexible office, organizational intervention, implementation, working environment, office design

## Abstract

Activity-based workplaces (ABW) have been implemented in many organizations to offer office flexibility and decrease facility costs. Evaluations of the ABW implementation process are rare. The study aimed to examine the ABW relocation process of two offices in a Swedish governmental agency and to explore factors that influence the implementation process and satisfaction with it. Qualitative or quantitative data were collected on process variables (context, recruitment, reach, dose delivered, dose received, satisfaction), barriers and facilitators to the process were explored in focus group interviews, and immediate outcomes (perceived knowledge, understanding office rules, satisfying information and support) were measured by questionnaire before and after the relocation. The evaluation showed that recruitment was unsatisfactory and reach insufficient—and participation in activities was thus low for both offices. However, intended changes improved. Unclear aims of ABW, lack of manager support and, lack of communication were some of the reported barriers to participation, while a well-planned process, work groups, and program activities were facilitators. Thus, to increase satisfaction with the relocation, our results suggest that recruitment should be thoroughly planned, taking these factors into account to increase participation. This knowledge may be useful for planning and designing successful ABW relocations and evaluations.

## 1. Introduction

As today’s working life becomes more digitalized and knowledge work more interactive, employees need to be able to communicate and interact with greater flexibility than before [1]. Organizations try to meet these working life changes by creating appropriate office environments that facilitate interaction among employees and the sharing of knowledge, with better technological solutions, lower facility costs, and increased flexibility for quickly implementing organizational changes [1,2,3]. In addition, the pandemic outbreak of COVID-19 resulted in a global increase in working from home [4], which may remain common even after the pandemic. This underlines the need to focus on flexible office solutions with respect to organizational changes as well as changes to society as a whole.

Establishing an activity-based workplace (ABW) scheme is a common office solution [5], which is characterized by open work spaces and no assigned work places, for employees who work to varying degrees both in and outside the office facilities. Further, ABWs aim to support work by providing a variety of work areas designed for different types of office work activities, such as those necessitating communication, collaboration, or concentration in particular [6].

Research focusing on the effects of implementing ABWs is growing, although the findings surrounding them are still ambiguous. Most studies have been on the effects of ABWs on individuals’ perceived satisfaction with, for example, performance [3,7,8,9,10,11,12], communication [12,13], and collaboration [13,14]. Satisfaction with spatial factors, such as office layout [8,10,13,15], desk sharing, and privacy [5,12,13,16] has also been studied. Switching behavior (i.e., between different types of work activity areas) has been of interest for determining whether it reduces sedentary behavior in ABW [5,17,18,19]. However, moderators that may explain ambiguous results concerning satisfaction (or lack of it) with ABW in relation to, for example, leadership [20] and task requirements [2] have received less attention. Moreover, evaluations of the implementation process and related factors of importance for satisfaction with ABW among employees and the organization are rare [21,22,23].

Most previous studies evaluating the impact of ABW on specific outcomes do not explain how or why the implementation of ABW was successful or not. Thus, there is little guidance on how to achieve a satisfying process. In addition, we found no studies applying theories for the process evaluation design and analyses [24]. Applying theories in process evaluations are necessary for researchers accommodating the need to provide or develop guidance for process evaluations in implementation of ABW [25]. Thus, the lack of process evaluations of ABW implementations calls for more research.

There is an agreement among researchers that an implementation does not just happen by itself, that it is a process focused on achieving beneficial outcomes for individuals and organizations [26,27]. Further, research indicates that the implementation process influences desired outcomes but needs to be carefully planned and executed [27,28,29]. In a review by Wierenga et al. [30], organizational implementations of worksite programs to improve employee health were characterized by a lack of systematically performed evaluations, and the barriers and facilitators were only documented and evaluated after and not during the implementation [30]. In addition, funders and companies are calling for implementation research that can serve to advance knowledge, develop methods, and provide information needed for organizations that are considering implementations, especially those on a larger scale [27]. Thus, as the interest in utilization of ABW increases, there is a growing need to evaluate the implementation process in order to understand not only if, but also how and why it can be successful in achieving the intended changes [31,32]. Further, organizations considering implementing ABW can benefit from further knowledge about the barriers and facilitators [31] that may influence the implementation process.

**Figure 1:**
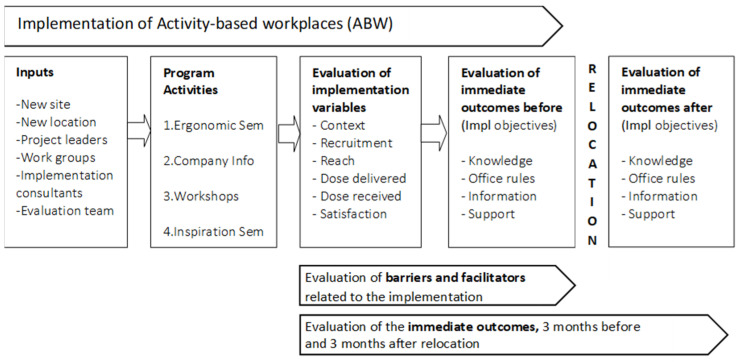
The logic model shows the process of implementation, resources (inputs), program activities and the evaluation of implementation variables and immediate outcomes in the present study.

Not all interventions are deliberately based on certain theories but they reflect assumptions regarding how actions will produce change [33]. Therefore, it is important to clarify these assumptions by constructing a logic model [33]. In the present study, we use a logic model to delineate the activities for producing change, potential causes of problems, and the immediate outcomes aimed for in the implementation (Figure 1). For example, if an ABW implementation includes a seminar on ergonomics for those affected, it may reflect an assumption among those in charge of the implementing that dissatisfaction with the implementation may be due to a lack of knowledge of how the implementation will affect the ergonomics of the workspace.

There is a diversity of frameworks for process evaluations [33] that evaluate whether interventions were implemented as intended, which can be useful for advancing our understanding of how and why an implementation works [34]. We used a framework developed by Linnan and Steckler [34], which utilizes six key process evaluation variables: context, recruitment, reach, dose delivered, dose received, and satisfaction.

The aim of this study was to evaluate implementations of ABW in two offices within the same organization, where the same implementation strategy was used for both offices. More specifically, we looked at whether the program activities were implemented as planned and if they led to the intended changes. Evaluating two offices made it possible to examine how potential differences between certain implementation variables (i.e., recruitment, reach, dose delivered, and dose received) or between the program activities may have impacted the intended changes. Further, to deepen our understanding of what might influence the implementation process variable of relocation, we aimed to determine what individual- and organizational-level factors acted as barriers and facilitators to this process. The following research questions were addressed:

(1) Was the relocation to ABWs, in two geographically separated offices within the same organization, implemented as intended with regard to the six process variables of context, recruitment, reach, dose delivered, dose received, and satisfaction [34]?
(2) To what extent did the implementation process accomplish the intended changes with regard to increased knowledge about ABW, understanding office rules, and satisfaction with information and support?
(3) What key factors, barriers, and facilitators, related to the implementation process, were perceived by the employees?

## 2. Materials and Methods

### 2.1. Background

This study observed how a large government agency (the Swedish Transport Administration) conducted the implementation of activity-based workplaces (ABW) at two office sites, office A (OA) and office B (OB), located in two cities in Sweden. This organization is a national agency with central and local administrations. Most employees have flexible working arrangements (such as flex-time or unregulated working hours), work remotely and in projects with shorter office meetings, or work via web-based meetings and occasional visits to different office sites [35]. According to the organization’s management, working in these ways requires new approaches to utilizing office space in order to meet employees’ needs and increase flexibility around office occupancy.

### 2.2. Design

The two offices relocated to ABWs in August 2018 (OA) and January 2019 (OB). Starting the process about one year prior to relocation, the organization employed an explicit, carefully planned implementation process involving managers and employees. While researchers were not involved with the planning and realization of the implementation, consultants were engaged to help to design and tailor the program activities as well as run some of the activities (Section 2.4.2). To evaluate the course of the process, the researchers systematically tracked the implementation through three rounds of data collection, comprised of two waves of questionnaires, before and after the relocation, and one round of group interviews prior to relocation. All participants signed an informed-consent form prior to participation. The study was approved by the Regional Ethical Review Board in Uppsala, Sweden (Dnr.2015/118).

### 2.3. Offices

The number of employees differed between the two offices, and the sizes of the spaces they were moving to were similar to their original office spaces (Table 1). The allocation of different types of working areas, that is, cell offices (private), shared rooms (2–3 persons), open-plan offices (≥4 persons), and other solutions, differed between the offices, as OA’s space included more open-plan solutions while OB’s space utilized more cell offices and shared rooms (Table 1). In the new ABWs, areas (defined as zones) for different types of work or ways to perform work were designated into active, middle, calm, and quiet zones. The allocation of zones was about the same for OA as for OB; however, OA had six floors while OB had two floors. The distribution of supporting work stations in the office, in the form of work places with two screens, meeting rooms, small rooms, telephone rooms, coffee corners, and prioritized seats for employees with special needs or physical, mental, or organizational needs (e.g., confidentiality), was about the same in both offices in relation to the number of employees (Table 2).

### 2.4. The ABW Implementation Process

#### 2.4.1. Implementation Objectives

Based on the organization’s rationale for the implementation strategy and its guidelines for implementation of ABWs, the project management highlighted four objectives to be accomplished during the implementation process: to increase employees’ knowledge about ABW, increase their understanding of the new office rules, facilitate the transparency of the process by providing satisfying information, and to provide support during the process. At both offices, the top management group assigned a project manager from within the organization to be in charge of the new office premises and the design of the activity-based workplace. They also established a work group including about ten representatives from different departments (planning, investments, central management, support, maintenance, purchase, logistics, and communication), which was assigned to prepare the employees for the new activity-based way of working. Four different program activities were planned for fulfilling the objectives of the implementation process (Section 2.4.2).

#### 2.4.2. Program Activities

All of the program activities were offered at the workplace during office hours and participation was voluntary. One of these activities, the modern ergonomics seminar, aimed to inspire interest in the activity-based way of working. It was offered twice for each office by the same expert consultant. The management information activity was concerned with the local implementation of ABW, addressing why and how to implement the ABW, and utilizing a questions and discussion approach, presented by organization management three times for OA and twice for OB. The workshops activity aimed to provide knowledge and tools for engaging in activity-based work. It was offered to OA on nine occasions and to OB on eight occasions. The fourth activity, the inspiration seminar, facilitated learning and understanding more about the changes happening in today’s working life. It also provided guidance on how to do activity-based work, focusing on why and how the agency is implementing ABW as well as the expectations, threats, and challenges to be faced as a manager in the new office. Presented by different expert consultants, the seminar was offered to OA on three occasions and to OB on two occasions.

### 2.5. Participants and Data Collection

#### 2.5.1. Questionnaires

All of the employees considered in the relocation to ABWs at the time it commenced (OA *n* = 776, OB *n* = 285) were invited to the study and received a link to a questionnaire three months before the relocation (baseline). Note that a number of employees who had used the office spaces in question were not invited to answer the baseline questionnaire, such as consultants, employees relocating to other offices, and employees moving in from other offices to the new ABW office. Therefore, the company data on the number of employees utilizing the office areas before and after relocations in Table 1 differ from the number of employees included in this study. The response rate was 62% (*n* = 481) for OA and 75% (*n* = 215) for OB. All of the employees working in the new ABWs (OA *n* = 586, OB *n* = 268) were invited to answer a three-months follow-up questionnaire, including employees moving in from other offices. The response rates were 70% (*n* = 412) and 82% (*n* = 220) for OA and OB, respectively. The number of employees to respond to both the baseline and the three-month follow-up questionnaires was 300 employees for OA and 176 for OB.

The distribution of women and men for OA was 46% women and 54% men, and the mean age (SD) was 45 (11); for OB it was 60% women, 40% men, and a mean age of 47 (10). Seven percent of employees in OA and twelve percent in OB were managers. Exclusion criteria for the study population were being on sick leave or parental leave and reporting a change in job or retirement.

#### 2.5.2. Semi-Structured Interviews

The project managers and work group members asked employees if they were interested in participating in focus group interviews. If they accepted, researchers sent them an invitation with the details for participation and time for the interview. Six focus group interviews were conducted among the employees of each office, two with the managers (OA *n* total = 11, OB *n* total = 10) and four with the other employees (OA *n* total = 21, OB *n* total = 21). The focus groups were mixed with regard to the departments the employees worked in (i.e., planning, investments, central management, support, maintenance, purchase, logistics, and communication).

The focus group interviews were semi-structured and held three months prior to the relocation. An interview guide was used to identify and discuss the barriers and facilitators to the implementation, addressing them on organizational, individual, and process-design levels in relation to the organization’s objectives. The interviews aimed to capture participants’ opinions in relation to certain themes, such as support from management (organization), meaningfulness and participation (individual), and transparency and information (process design). To identify unexpected aspects, an open question about the factors that hinder or facilitate the process was included. All participants signed an informed-consent form. Interviews were recorded and had a maximum duration of 60 min.

### 2.6. Evaluation

#### 2.6.1. Evaluation of Implementation Variables

The evaluation of the implementation of ABW focused on the delivery of the program activities and followed a framework comprised of six variables recommended by Linnan and Steckler [34]: *context, recruitment, reach, dose delivered, dose received*, and *satisfaction*.

*Context* relates to physical environmental factors that may influence satisfaction with the implementation of ABW; information on this was provided by the local project manager and by organizational records. The *recruitment* variable concerns how participants were recruited to different program activities, as described by the local project manager. *Reach*, to what extent employees attended different program activities, was assessed in the questionnaire with the response alternatives “yes” or “no” for each activity. The variable of *dose delivered* refers to the number of opportunities employees had to attend different program activities, and data on this were gathered from the organization. *Dose received* relates to the extent to which participants felt that they had the opportunity to participate in the process and was captured in the baseline questionnaire using a six-point scale from 1 (“not at all”) to 6 (“to a large extent”). Also related to this variable is the question of whether participants were engaged in the process by, for example, reading information about it, asking questions, and attending program activities was evaluated using a six-point scale from 1 (“not at all”) to 6 (“to a large extent”).

The *satisfaction* variable regards whether the program activities were satisfying in terms of their perceived relevancy, increased knowledge received, content delivered, and whether they led to changed attitudes and decreased worries; these aspects were measured on a six-point scale 1 (“not at all”) to 6 (“to a large extent”) in the baseline questionnaire.

#### 2.6.2. Evaluation of Immediate Outcomes

The immediate outcomes, *knowledge, office rules, information, and support*, were each measured with a single item, both before and after the relocation, with the overall purpose of capturing employees’ views on the implementation process and on what the organization’s objectives are with the implementation process. The questions were “to what extent do you...” “receive the knowledge you need about ABW to feel confident?” *(knowledge)*, “begin to understand the new office rules?” *(office rules)*, “receive the information you need at the right point of time?” *(information),* and “know who to address your questions to?” *(process support).* Questions were rated on a six-point scale from 1 (“not at all”) to 6 (“to a very large extent”).

#### 2.6.3. Evaluation of Barriers and Facilitators

Existing models for assessing barriers and facilitators to implementations have mainly been designed for healthcare settings and were therefore not deemed to be applicable for the present study. Looking a bit further afield to management and employee training research, we found the transfer of training model by Baldwin and Ford to provide a better framework; it is based on the idea that the outcome of a program is dependent on the organization, individual characteristics, and the training design [36]. We used a modified version of this model to identify and categorize barriers and facilitators to the implementation process, based on whether they were on the organizational level, relating to support from management; on the individual level, in terms of employees perceived meaningfulness and participation; or on the process design level, relating to transparency and informativeness. Certain verbal expressions from the interviews were classified as pertaining to each of these categories.

### 2.7. Data Analyses

Quantitative data were analyzed using IBM SPSS Statistics 27 (IBM, Armonk, NY, USA). Descriptive statistics of questionnaire data were derived using frequencies and percentages for categorical variables and means and standard deviations (SD) for categorical and continuous variables. The process variables of context, recruitment, and dose delivered were described using interview data. Reach, dose received, and satisfaction were dichotomized to present the proportions of employees reporting high satisfaction values (i.e., 5 and 6 on the six-point scale, corresponded to “to a large extent”). Changes in immediate outcomes *(knowledge, office rules, information, and support)* were analyzed using repeated measures ANOVA with time (3 months before and 3 months after) as a within-subject factor, office (OA vs. OB) as a between-subject factor, and including the interaction of office and time.

All interviews were recorded and transcribed verbatim. Qualitative content analysis [37] was used to analyze the interviews. Two researchers independently read the text before they met to reach a consensus. They then extracted and coded the barriers and facilitators found in the text and categorized them into the corresponding categories and subcategories of an organizational level (support from management, individual level (meaningfulness, participation), and process design level (transparency, information)).

## 3. Results

### 3.1. Evaluation of the Process Evaluation Variables of Context, Recruitment, Reach, Dose DELivered, Dose Received, and Satisfaction

*Context* (i.e., factors relating to the physical environment)—A project manager and a working group were recruited by the organization’s top management for both offices. The implementation process started for both offices in the beginning of 2018, with OA moving seven months later and OB eleven months later. OA moved to a building that was about the same distance to the city center as their previous location but closer to commuting accessibility. OB moved to a building with a longer distance from the city center and less commuting accessibility. While both offices had five floors before relocation, OA moved to six floors and OB to two floors. OA had a somewhat smaller office area per employee after the relocation (OA 13 m^2^, OB 15 m^2^), and proportionally less web-meeting space (OA 7%, OB 14%) and less small meeting space (OA 3%, OB 6%), compared with OB (Table 1 and Table 2).

*Recruitment*—Information and invitations to participate in program activities were posted on the intranet and indoor posters. According to the interviews, employees were informed about the activities from their colleagues and attending was optional for employees of both offices. Both managers and employees expressed their concern about activities being optional.

*Reach*—Less than half of the employees participated in the program activities except for a higher participation in the workshops for OB. Participation for both employees and managers was in general lower for OA than for OB. The proportions of employees not attending any of the activities was also higher for OA than OB, with 48% of OA (non-managerial) employees not attending versus 15% for OB, and the corresponding percentages of 47% and 8% among the managers (Table 3).

*Dose delivered*—The number of program activities offered and their content were similar for both offices. OA (with more than twice as many employees) was offered one more modern ergonomics seminar and one more workshop.

*Dose received*—For dose received the results show that the employees of both offices expressed having very little opportunity to participate (OA 12%; OB 17%) and engaged in the process only 22%. However, the results for managers show that about half of the managers perceived a high level of opportunity to participate (OA 50%; OB 48%) and engage in the process by taking in information, communicating questions, and attending program activities (OA 65%; OB 52%).

**Figure 2:**
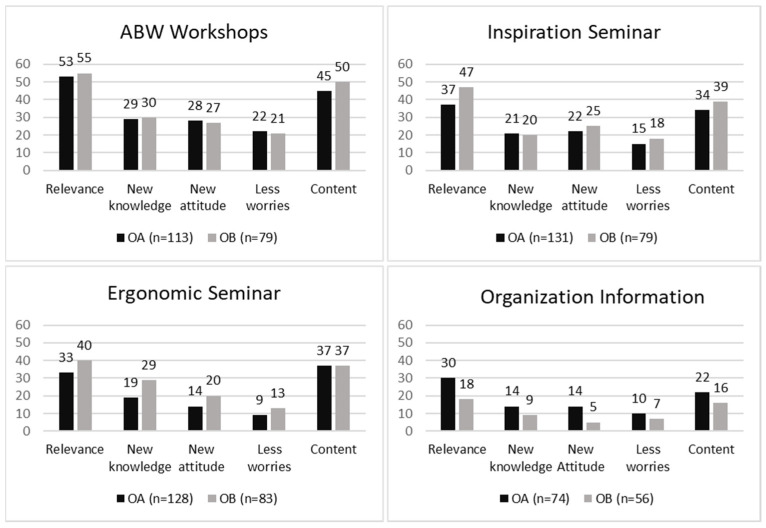
The percentage of employees reporting high satisfaction with the aspects of relevance, increased knowledge, changed attitude, decreased worries, and content in the program activities.

*Satisfaction* with program activities—The proportions of employees reporting they were satisfied to a large extent with the evaluated aspects of the activities varied for the different activities, between 9% and 53% for OA and between 5% and 55% for OB. For both offices, the percentages of participants reporting high satisfaction with the aspects of relevance and content for the different activities were greater than those for increased knowledge, changed attitude, and decreased worries (Figure 2). For ABW workshops, the proportions of participants in both offices reporting high ratings of satisfaction with all five aspects were higher than the corresponding proportions for the other activities (Figure 2).

In summary, the implementation seemed to have been carried out as planned with respect to the physical environmental context factors and the dose delivered of different activities. However, recruitment by using established channels, intranet, and posters did not seem successful since reach was low; indeed, less than half of the employees had participated in the various program activities, and only three out of ten participated in one of the four activities. Further, dose received, regarding satisfaction with the opportunity to participate or actually engage in the process, was low and less than half of the participants reported high satisfaction with the evaluated aspects of the activities. In general, the offices showed no major differences regarding their evaluations of the implementation variables under study.

### 3.2. Changes in the Immediate Outcomes (Knowledge, Office Rules, Information, and Support)

Among the employees participating in the relocations, the reported degrees of satisfaction with knowledge, office rules, information and support differed before and after the relocation. The mean values for all of these outcomes were already somewhat high for the first wave, range 3.85–4.16 (OA) and 4.27–4.40 (OB) (rated on a six-point scale). 

**Figure 3:**
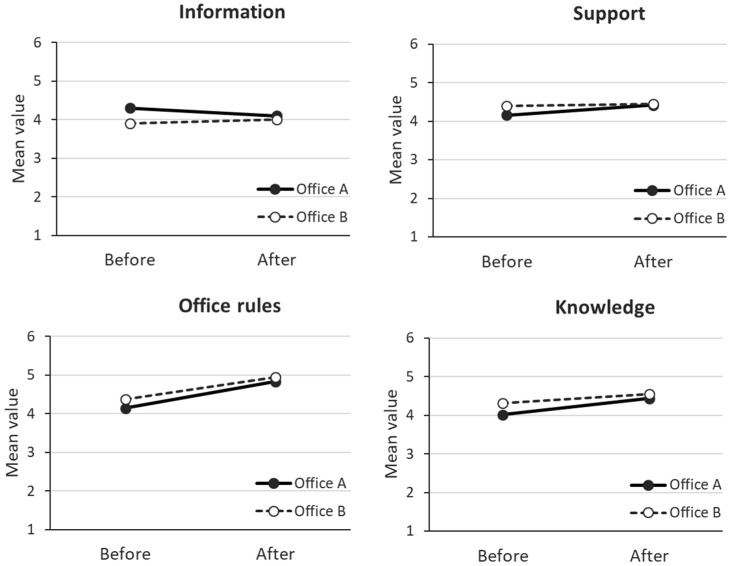
The mean values among Office A and B for satisfaction with perceived knowledge, understanding office rules, information, and support before and after relocation.

The mean values increased between the two measurement points for office rules (F (1477) = 124; *p* < 0.001), knowledge (F (1477) = 28.6; *p* < 0.001), and support (F (1477) = 4.8; *p* = 0.029) for both offices (Figure 3). Although there was a minor difference between the offices in regard to the mean values for information, with OA being higher and OB lower after the relocation (interaction: F (1477) = 4.57; *p* = 0.030), the overall mean values for this outcome did not change significantly (Figure 3).

In summary, satisfaction with knowledge about ABW, understanding office rules, support, and information (not OB) were higher after relocation, indicating that the implementation process may have the potential to influence satisfaction with the intended changes.

### 3.3. Perceived Barriers and Facilitators to the Implementation Process

#### 3.3.1. Support from Management

In the interviews employees of both offices described lack of support from management as a barrier to participation, although there was some understanding for why this was difficult for those managers who work remotely and lack proper information from top management. *“In an organization like this, a great responsibility is on you, at least that is why I have participated in the seminars, to get as much information as possible, because I don’t get it from my manager”*(OA employee). The managers among the participants agreed with this assessment, and confirmed they had lessened their role in the process and expected employees to take their own responsibility. One OA manager expressed it like this: *“messages from co-workers to co-workers is a greater strength”*. However, all groups discussed and agreed that managers are important for facilitating participation in the activities, especially since they were not mandatory.

In the interview discussions for both offices, the local project leaders and work groups were identified as facilitators for perceived support during the process. The work groups were seen as important for communication and perceived opportunity to participate. It was pointed out that work group members did not have allocated times for their work, which may have hindered their engagement.

#### 3.3.2. Meaningfulness and Participation

Unclear aims and incentives for the ABW implementation were brought up by both offices as barriers to participation. An OA employee shared how having an explicit implementation process is very important for meaningfulness, *“It is obvious that they have a strategy for the implementation, the only thing I miss is the aim; why they have chosen activity-based, it is not clear”*. The reasons that employees gave for not being interested in participating and committing time to the implementation were that employee involvement came late in the process (OA), that decisions had already been made and could not be changed (OA, OB), and that they had knowledge about the previous failures of implementing ABW (OB). According to an OB employee, *“I separate information and participation; information has been good I think, but again, it is only information about what is already decided above our heads; I still cannot say that they listen to us and our criticism”*.

#### 3.3.3. Process Design and Communication

Lack of feedback and dialogue with the work groups and managers as well as their not taking action on questions and ideas posed to them were described as barriers to a transparent process. However, this was less of an obstacle for employees with colleagues on the organization’s steering group, in the work groups, or who worked closely with the project leader (OA). For an OB employee, receiving information too early in the process created concerns about what will happen and when, *“this extended planning phase has been too drawn-out, some feel they don’t know how it is going to be; it’s better to just move, as now there is a lot of speculation”*. In contrast, early information was never an issue for OA, who described receiving too little and too late information as barriers.

Seminars and workshops facilitated process transparency and information sharing according to the interviewees. However, employees expressed that they were surprised that participating in the activities was optional and suggested that they should have been made mandatory. The use of different types of information (e.g., workshops, intranet, posters) was brought up as positive (OB) and both offices emphasized that the inspiration seminars and workshops were very important, *“the workshop made things clear; you could ask the project leader questions and the external experts talked about changing behavior; recommended for everyone, should have been mandatory though”* (employee OA). On the subject of transparency, one manager clarified the difficulties in finding information on the intranet and from the organization *“this relocation has been very well planned, there has been access to as much information as possible, but the problem has been how and where to get it”* (manager OA).

## 4. Discussion

We observed the implementation of activity-based workplaces (ABW) in two offices within a large government organization in Sweden. To our knowledge, this is the first study to systematically follow and evaluate the process, immediate outcomes, and barriers and facilitators of a relocation from traditional offices (with cell and shared rooms) to activity-based workplaces. The roles of program activities and participation for a successful implementation were explored and compared between the offices. The first aim was to explore whether the implementations of ABW were carried through as intended, in terms of whether the employees attended the activities and participated in the process as planned, and whether the implementation process led to intended changes [32], which was to increase knowledge about ABW, to instill an understanding of the office rules, and to evoke satisfaction with the information and support received. The qualitative data that we analyzed from the group interviews shed light on the factors that hindered or facilitated the realization of the intended changes (Table 4).

### 4.1. The Implementation Process

We found that the implementation and the program activities had been carried out as planned for both offices. However, reach, in terms of recruitment effectiveness, was less effective, as was indicated by the low levels of employee participation in the process and in the different program activities for both offices. This is in agreement with other studies indicating limited involvement in the process to be critical for a successful implementation [6,21,23]. The interviewees who had attended the program activities thought they should be mandatory and that employees would have prioritized participation if they had been better informed about the activities.

For dose received, in terms of the perceived opportunity to participate in the process, around only 15% of the employees perceived it to be high and only two out of ten employees reported actually engaging in the process (e.g., by reading information, attending activities). Since employees do not passively “receive” interventions but interact with them, the term “dose received” within the framework of Steckler and Linnan [34] has been used. Hence, to reach a better understanding of why and how employees do or do not respond to and interact with an implementation, we assessed the barriers and facilitators to it using qualitative data from focus group interviews along with quantitative data gathered from questionnaires [38]. Further, since the influence of interventions may differ due to contextual factors prior to or during the implementation, we studied two offices within the organization over time in order to explore factors that may strengthen or impede the results of the implementation.

In regard to the variable of context, the implementation phase took about seven months for OA and about eleven months for OB. No major differences were found for this variable between the offices, although, according to the interviews, the extended length of this phase for OB brought on speculations and worries that acted as a barrier to participation. However, OB could also take advantage and be inspired by positive reports and experiences from OAs relocation. Knowledge about previous implementation failures in other offices, via colleagues and the intranet, and the cynicism it gave rise to, known as “initiative fatigue” [39], created a barrier to wanting to and actually participating in the implementation. To prevent “initiative fatigue” from hindering participation, it needs to be counteracted prior to the implementation process, in part by presenting positive and inspiring reports about implementations on the intranet.

To try to ensure recruitment effectiveness (reach) and employee participation, the implementation used local project managers and work groups, and did not give line managers a pronounced role in the process. According to the interviews, participation in the program activities was optional and considered a barrier to recruitment and employee involvement—a view that suggests that it is not only the managers who influence employee participation. Although the employees were humble, showing an understanding of the challenges managers faced when working remotely, the interviewees argued that there is a need for line managers to support the relocation process and advocate participation, especially when the activities are optional. This expressed need for line managers to commit to supporting the process in these ways corresponds to research about the importance of manager involvement and support for successful organizational changes [39,40]. Line managers responsibility for employee involvement in organizational interventions has been associated with positive outcomes in previous studies [39], and a review by Nielsen [41] acknowledges the important role of line managers in the participatory process and in maintaining employees’ well-being throughout the organizational changes. Lahtinen [42] highlights the impact of exercising active leadership in the process in a study about relocation from traditional cell offices to ABW. Thus, there is a good deal of evidence suggesting that future relocations to ABW would likely be more successful at recruitment and participation if line managers were given an expanded role that involved better preparation and training regarding managing and supporting employee engagement in the process.

Another simple but practical reason for low participation occurring may relate to time constraints. The most attended activity was the workshops for OB (68%), which was offered repeatedly, on nine (OA) and eight (OB) occasions, during the implementation, to make it easier to fit it into a busy schedule. Still, only 25% participated in workshops among OA; however, since this office was larger, and employees may not have heard about the workshops from colleagues to the same extent as for OB, participation interest may have been stunted.

### 4.2. Satisfaction with the Process

In this study, unclear aims with the relocation to ABW was reported to be a barrier to participation, which is in line with the relocation study by Lahtinen [42] in which having clear goals and communication were found to be important for satisfaction with the process. In a previous study by our research team investigating the importance of indicators for sense of coherence during relocation the to ABW, not only was perceived meaningfulness, as an indicator, found to be important for satisfaction with the activity-based work environment, but participation in the program activities was found to then facilitate meaningfulness [40]. Since perceived meaningfulness makes changes easier to accept [43], this further emphasizes the importance of participation.

According to the interviews, the communication was insufficient due to inadequate feedback and lack of action on questions and ideas from the work group. This was a barrier to further participation and involvement for employees in both offices. In line with our results, a study by Brunia [21], comparing the best and worst cases of ABW implementations, found satisfaction with the process to be low due to a lack of involvement, work environment problems not being properly addressed, and a lack of guidance on how to properly use the ABW. The opposite, high satisfaction, was found among those sufficiently informed about ABWs—in other words, those who had been involved in the workshops on the new way of working and who had taken an active part in office design and arranging the time schedules for relocation [21].

In our study, despite the unclear aims and insufficient communication reported, the employees recognized an explicit implementation strategy for the relocation, referring to a “well-planned process” with a project leader, work group, and activities as facilitators. However, these facilitators did not seem to improve communication and clarify aims, which seems to be highly relevant to consider in future relocations.

### 4.3. Changes in the Immediate Outcomes

Despite some dissatisfaction with the process reported by the participants, participation showed an increase in knowledge about ABW and office rules, information, and support three months after relocation. However, these ratings were relatively high before relocation, which may be due to participation in program activities having likely influenced the employees’ personal awareness of these factors. The program activities per se may have the potential to increase satisfaction with ABW among employees. Future research should focus on elucidating the factors or activities that actually influence the intended outcomes in terms of understanding ABW, the office rules, and the new way of working as well as inspiring satisfaction with the process. These results further show the importance of recruitment and reach for having greater participation among employees, and thus greater satisfaction, which was pointed out in the interviews. Further, this study itself demonstrates the value of evaluating implementations, and its usefulness for revealing the process facilitators and barriers that need to be better understood in order for future ABW implementations to be more successful for all involved.

A minor difference between OA and OB was the higher participation in activities for OB. This may have influenced the ratings of the immediate outcomes, which were in general somewhat higher for OB before the relocation. However, these ratings were higher for both offices after the relocation, with OA’s ratings for information and support being even higher than OB’s. It is plausible that this difference may be due to the OB employees’ greater knowledge and expectations before the relocation increasing their expectations about the information and support they would receive after the relocation. Not surprisingly, understanding of the office rules was the outcome that increased the most after relocation, and one may believe that a new working concept like ABW probably becomes easier to understand when it is adopted into a real work setting.

### 4.4. Strength and Limitations

In this kind of empirical study there is always a challenge for the research team to get full access to the target of research and to describe the strategies and the planned implementation process to enable reproducibility. A strength with the present study is that it is based on a close collaboration with the organization, which enabled us to collect an extensive amount of longitudinal data with a mixed method approach. Further, the immediate outcomes could be measured at two different time points to observe changes over time. Another strength was using a framework of six process variables [34] and a framework for evaluating barriers and facilitators [36], which enabled a systematic evaluation that increased the quality of the evaluation [30,33]. Also, the process variables that we used will make it easier for other researchers of ABW implementations to compare with or replicate our research for other organizations.

Among the limitations of the study is that contextual factors were only taken into limited account when following the changes made by the organization. The fact that the two offices were part of the same organization restricts the generalizability of the results. Further, since the participants in the interviews were self-selected, they may have been somewhat more positive to the ABW concept than non-participants, and thus in this respect not representative of all employees. This, however, should not have been particularly impactful since the interviews aimed to gather deeper understandings about a variety of implementation aspects.

### 4.5. Practical Implications and Future Research

We want to encourage researchers to perform process evaluations of ABW implementations and apply theories for a better understanding. The findings of our study contribute with knowledge about the importance of the implementation process to accomplished intended changes and describe how the implementation work and why. It appeared in our study that the process activities to some extent attained intended changes among participants, which indicate a satisfying implementation. However, since participation was low, changes were limited and satisfaction with the relocation not fulfilled. Thus, further evaluation of process variables could determine that recruitment and thus reach was insufficient, motivating the evaluation of process barriers and facilitators.

For researchers our study applying theories [34,36] can provide a resource for planning data collections and analyses. Theory-based process evaluations can contribute to guidance on how to design, plan and implement ABW, which are needed to help understanding the process and core barriers and facilitators to intended outcomes [24]. Further, in collaboration with organizations, research findings can facilitate development of the evaluated intervention.

For organizations to obtain a satisfying implementation process of ABW, based on our findings we recommend to design the relocation applying a logic model to clarify the theory and the process for how program actions will produce change. Early in the planning phase, employees should be involved and the framework variables recruitment and reach addressed, since they appear to be associated with employee participation. Moreover, organizations should target the identified facilitators, i.e., use workgroups, program activities and management support to facilitate participation and communication during the process.

The use of theories in future implementation research is desirable [24] and we suggest that future research should further evaluate ABW implementation processes applying theories, and explore critical aspects for successful implementations, e.g., adoption to the ABW and “another way of working”.

## 5. Conclusions

The way in which the implementation process for relocating to ABW took place seemed to impact whether the intended changes occurred regarding knowledge about ABW, understanding the office rules, and satisfaction with information and support. This study found that satisfaction regarding knowledge, office rules, information, and support changed as intended after employees relocated to ABW. However, the evaluation revealed that, for both offices, participation in the activities was low, satisfaction with recruitment was low, and reach was thus insufficient. In addition, unclear aims of ABW, lack of manager support and lack of communication were reported barriers to participation, while a well-planned process, work groups, and program activities were facilitators. These findings suggest that to increase satisfaction with a relocation, recruitment should be thoroughly planned in order to increase participation and thus satisfaction. This knowledge can be applied when deciding whether to undertake such an implementation as well as when planning and designing ABW relocations and evaluations.

## Figures and Tables

**Table 1:** Description of office parameters (data based on company reports) in office A (OA) and office B (OB) before and after relocation to ABW. Office area given as a total and per employee (in parentheses).

Office	Employees (*n*)	Office Area	Floors	Office Type Cell/Shared/Open-Plan/Other
OA (before relocation)	825	15 704 (19)	5	32%/11%/41%/16%
OB (before relocation)	275	5 172 (19)	5	47%/40%/2%/11%
				Allocated area for work zones active/middle/calm/quiet
OA (after relocation)	1087	14 248 (13)	6	50%/20%/20%/10%
OB (after relocation)	338	5 065 (15)	2	48%/28%/18%/6%

**Table 2:** Supporting work places, rooms, coffee corners, and canteen seats in OA and OB after relocation to ABW. The percentages relate to the total number of employees in the office.

	Work Places (Tables) with Two Screens	Web-Meeting Rooms	Meeting Rooms	Small Rooms	Telephone Rooms	Coffee Corners	Canteen Sea	Prioritized Seats
OA Employees *n* = 1087	670 (62%)	77 (7%)	60 (6%)	31 (3%)	4	15	350 (32%)	30 (3%)
OB Employees *n* = 338	229 (68%)	49 (14%)	19 (6%)	22 (6%)	2	3	130 (38%)	3 (1%)

**Table 3:** The numbers and percentages of employees and managers who participated in program activities before the relocations of the two office sites.

Reach	Office A		Office B	
	Employees *n* = 449 *n* (%)	Managers *n* = 34 *n* (%)	Employees *n* = 190 *n* (%)	Managers *n* = 25 *n* (%)
Modern Ergonomics	28 (28%)	5 (15%)	83 (44%)	13 (52%)
Management information	74 (16%)	7 (21%)	56 (30%)	8 (32%)
ABW Workshops	113 (25%)	8 (24%)	129 (68%)	16 (64%)
Inspiration Seminar	131 (29%)	9 (26%)	79 (42%)	9 (36%)
Number of attended activities				
0	214 (48%)	16 (47%)	28 (15%)	2 (8%)
1	99 (22%)	12 (35%)	52 (27%)	8 (32%)
2	81 (18%)	2 (6%)	58 (30%)	7 (28%)
3	35 (8%)	3 (9%)	29 (15%)	8 (32%)
4	20 (4%)	1 (3%)	23 (12%)	0

**Table 4:** A summary of implementation barriers and facilitators related to the organization, individual, and process design.

	Facilitators to the Process	Barriers to the Process
Organizational level	Local project managers and work groups	Lack of support from management
Individual level	An explicit implementation process Early involvement	Unclear aims and incentives Late involvement, decisions already made Too early involvement Knowledge about previous failure
Process design level	Activities as seminars and workshops Information through different channels	Lack of feedback, dialogue and action on questions and ideas from managers and work groups Lack of timely information Optional participation in activities Difficult to find information

## Data Availability

Data are available upon reasonable request.
